# Supplementary material for: Stiffness and tension gradients of the hair cell’s tip-link complex in the mammalian cochlea
Source: eLife. 2019 Apr 1;8:e43473. doi: 10.7554/eLife.43473 (PMC6464607; doi:10.7554/eLife.43473)
Supplement: Figure 2—source data 1. — The table lists p-values resulting, respectively, from a one-way ANOVA to assay statistical significance of the measured mean-value variation of the hair-bundle stiffness ΚHB between different cochlear locations for inner (IHC) and outer (OHC) hair cells, from two-tailed unpaired Student's t-tests with Welch’s correction to compare mean values of ΚHB between two groups of a given hair-cell type (IHC or OHC) with different characteristic frequencies (CF) or between the two cell types (OHC/IHC) when they are associated to the same characteristic frequency. The last entry (Gradient OHC vs. gradient IHC) provides the p-value to assay the statistical significance between the slopes of a weighted linear regression of the relation between ΚHB and the characteristic frequency of the hair cell. A bold font was used to help find statistically significant differences. [file elife-43473-fig2-data1.docx]

|  | **ANOVA** | | **IHC** | | | | | |
| --- | --- | --- | --- | --- | --- | --- | --- | --- |
|  | IHC | OHC | 1-2 kHz | 1-4 kHz | 1-15 kHz | 2-4kHz | 2-15 kHz | 4-15 kHz |
| $K_{\mathrm{HB}}$ | ******p* =**  **5.0×10^-13^** | ******p* =**  **1.7×10^-15^** | *p* =  3.8×10^-1^ | ******p* =**  **6.4×10^-5^** | ******p* =**  **3.1×10^-8^** | ******p* =**  **1.5×10^-4^** | ******p* =**  **1.8×10^-7^** | *****p* =**  **5.1×10^-3^** |
|  | **OHC** | | | **OHC/IHC** | | | Gradient OHC *vs.* gradient IHC | |
|  | 1-2 kHz | 1-4 kHz | 2-4 kHz | 1 kHz | 2 kHz | 4 kHz |  |  |
| $K_{\mathrm{HB}}$ | *****p* =**  **1.7×10^-3^** | ******p* =**  **9.2×10^-12^** | ******p* =**  **2.7×10^-9^** | *****p* =**  **7.4×10^-3^** | ******p* =**  **4.4×10^-5^** | ******p* =**  **4.5×10^-9^** | ****p* =  1.8×10^-2^** | |

**Figure 2‒source data 1: Statistical significance.**

The table lists p-values resulting, respectively, from a one-way ANOVA to assay statistical significance of the measured mean-value variation of the hair-bundle stiffness $K_{\mathrm{HB}}$ between different cochlear locations for inner (IHC) and outer (OHC) hair cells, from two-tailed unpaired Student's *t*-tests with Welch’s correction to compare mean values of $K_{\mathrm{HB}}$ between two groups of a given hair-cell type (IHC or OHC) with different characteristic frequencies (CF) or between the two cell types (OHC/IHC) when they are associated to the same characteristic frequency. The last entry (Gradient OHC *vs.* gradient IHC) provides the p-value to assay the statistical significance between the slopes of a weighted linear regression of the relation between $K_{\mathrm{HB}}$ and the characteristic frequency of the hair cell. A bold font was used to help find statistically significant differences.
