# Supplementary material for: Stiffness and tension gradients of the hair cell’s tip-link complex in the mammalian cochlea
Source: eLife. 2019 Apr 1;8:e43473. doi: 10.7554/eLife.43473 (PMC6464607; doi:10.7554/eLife.43473)
Supplement: Figure 3—source data 1. — Data correspond to mean values ± SEM, with the number of cells indicated in parentheses. The width W and height h of the hair bundle are used to calculate an effective hydrodynamic radius RHB (see Equation (1) in Materials and methods) and plotted as a function of the hair cell’s characteristic frequency in Figure 3—figure supplement 2. [file elife-43473-fig3-data1.docx]

|  | Characteristic frequency (kHz) | 1 | 2 | 4 | 15 |
| --- | --- | --- | --- | --- | --- |
| Inner hair cells | Width $W$ (µm) | 9.3 ± 0.2 (n=11) | 9.5 ± 0.2 (n=6) | 9.4 ± 0.2 (n=17) | 9.2 ± 0.1 (n=8) |
|  | Height $h$ (µm) | 3.8 ± 0.2 (n=10) | 3.7 ± 0.1 (n=10) | 3.6 ± 0.2 (n=10) | 3.3 ± 0.1 (n=6) |
|  | Number of stereocilia $N_{\mathrm{SP}}$ | 46.6 ± 1.1 (n=10) | 56.4 ± 0.6 (n=10) | 59.0 ± 0.8 (n=10) | 66.6 ± 0.9 (n=10) |
|  | Effective radius $R_{\mathrm{HB}}$ (µm) | 2.9 | 2.9 | 2.9 | 2.7 |
| Outer hair cells | Width $W$ (µm) | 5.2 ± 0.1 (n=19) | 5.7 ± 0.1 (n=10) | 6.3 ± 0.1 (n=13) | N.A. |
|  | Height $h$ (µm) | 5.3 ± 0.5 (n=4) | 4.8 ± 0.2 (n=10) | 4.1 ± 0.2 (n=10) | N.A. |
|  | Number of stereocilia $N_{\mathrm{SP}}$ | 52.0 ± 2.0 (n=10) | 57.0 ± 1.3 (n=10) | 70.6 ± 1.7 (n=10) | N.A. |
|  | Effective radius  $R_{\mathrm{HB}}$ (µm) | 2.6 | 2.6 | 2.5 | N.A |

**Figure 3‒source data 1: Morphological parameters of inner and outer hair-cell bundles.**

Data correspond to mean values ± SEM, with the number of cells indicated in parentheses. The width $W$ and height $h$ of the hair bundle are used to calculate an effective hydrodynamic radius $R_{\mathrm{HB}}$ (see Eq. 1 in Methods) and plotted as a function of the hair cell’s characteristic frequency in Figure 3‒figure supplement 2.
