# Supplementary material for: Stiffness and tension gradients of the hair cell’s tip-link complex in the mammalian cochlea
Source: eLife. 2019 Apr 1;8:e43473. doi: 10.7554/eLife.43473 (PMC6464607; doi:10.7554/eLife.43473)
Supplement: Figure 3—source data 2. — The table lists p-values resulting, respectively, from a one-way ANOVA to assay statistical significance of the measured mean-value variation of a given variable between different cochlear locations for inner (IHC) and outer (OHC) hair cells, from two-tailed unpaired Student's t-tests with Welch’s correction to compare mean values of the variable between two groups of a given hair-cell type (IHC or OHC) with different characteristic frequencies (CF) or between the two cell types (OHC/IHC) when they are associated to the same characteristic frequency. The last entry provides the p-value to assay the statistical significance between the slopes of a weighted linear regression of the relation between the variable and the characteristic frequency of the hair cell. A bold font was used to help find statistically significant differences. The variables in the table correspond to the relative contribution r and the absolute contribution ΚGS of the gating springs to the hair-bundle stiffness, the contribution ΚSP of the stereociliary pivots to the hair-bundle stiffness, the rotational stiffness κ of a single stereocilium, and the stiffness kGS of a single gating spring. [file elife-43473-fig3-data2.docx]

|  | **ANOVA** | | **IHC** | | | | | |
| --- | --- | --- | --- | --- | --- | --- | --- | --- |
|  | IHC | OHC | 1-2 kHz | 1-4 kHz | 1-15 kHz | 2-4kHz | 2-15 kHz | 4-15 kHz |
| $r$ | *p* =  7.6×10^-2^ | ****p* =**  **2.0×10^-2^** | *p* =  5.3×10^-2^ | *p* =  1.2×10^-1^ | *p* =  9.2×10^-2^ | *p* =  3.2×10^-1^ | *p* =  4.3×10^-1^ | *p* =  7.4×10^-1^ |
| $K_{\mathrm{GS}}$ | ******p* =**  **1.3×10^-5^** | ******p* =**  **1.7×10^-6^** | ****p* =**  **2.1×10^-2^** | ******p* =**  **8.9×10^-5^** | ******p* =**  **3.3×10^-6^** | ****p* =**  **4.2×10^-2^** | ******p* =**  **4.4×10^-4^** | ****p* =**  **3.9×10^-2^** |
| $K_{\mathrm{SP}}$ | ******p* =**  **2.0×10^-14^** | ******p* =**  **8.3×10^-8^** | *p* =  6.6×10^-1^ | ******p* =**  **1.9×10^-4^** | ******p* =**  **1.5×10^-8^** | ******p* =**  **3.6×10^-5^** | ******p* =**  **1.3×10^-8^** | *****p* =**  **4.1×10^-3^** |
| $\kappa$ | *****p* =**  **1.0×10^-3^** | *p* =   7.7×10^-1^ | *p* =  1.6×10^-1^ | *p* =  7.0×10^-2^ | ****p* =**  **1.4×10^-2^** | *****p* =**  **1.6×10^-3^** | ******p* =**  **3.9×10^-5^** | *p* =  7.3×10^-1^ |
| $k_{\mathrm{GS}}$ | ****p* =**  **3.0×10^-2^** | *****p* =**  **1.9×10^-3^** | *p* =  3.0×10^-1^ | *****p* =**  **1.6×10^-3^** | *****p* =**  **1.8×10^-3^** | ****p* =**  **1.5×10^-2^** | ****p* =**  **1.6×10^-2^** | *p* =  1 |
|  | **OHC** | | | **OHC/IHC** | | | Gradient OHC *vs.*  gradient IHC | |
|  | 1-2 kHz | 1-4 kHz | 2-4 kHz | 1 kHz | 2 kHz | 4 kHz |  |  |
| $r$ | *p* =  5.8×10^-1^ | ****p* =**  **1.3×10^-2^** | *p* =  5.5×10^-2^ | *p* =  2.7×10^-1^ | *p* =  5.3×10^-1^ | ****p* =**  **2.1×10^-2^** | ****p* =  2.8×10^-2^** | |
| $K_{\mathrm{GS}}$ | *p* =  1.5×10^-1^ | ******p* =**  **2.2×10^-4^** | ******p* =**  **7.1×10^-4^** | ****p* =**  **1.4×10^-2^** | *p* =  1.5×10^-1^ | ******p* =**  **7.0×10^-4^** | *p* =  1.6×10^-1^ | |
| $K_{\mathrm{SP}}$ | ****p* =**  **1.8×10^-2^** | ******p* =**  **2.1×10^-5^** | ******p* =**  **9.5×10^-4^** | ****p* =  3.9×10^-2^** | ******p* =**  **5.2×10^-5^** | ******p* =**  **9.1×10^-4^** | *****p* =  7.6×10^-3^** | |
| $\kappa$ | *p* =  6.6×10^-1^ | *p* =  4.8×10^-1^ | *p* =  6.6×10^-1^ | *****p* =**  **8.1×10^-3^** | ******p* =**  **1.4×10^-9^** | *****p* =**  **6.1×10^-3^** | *p* =  1.9×10^-1^ | |
| $k_{\mathrm{GS}}$ | *p* =  6.9×10^-1^ | *****p* =**  **6.1×10^-3^** | *****p* =**  **8.5×10^-3^** | *p* =  8.1×10^-2^ | *p* =  6.3×10^-2^ | ****p* =**  **1.5×10^-2^** | *p* =  1.5×10^-1^ | |

**Figure 3‒source data 2: Statistical significance.**

The table lists p-values resulting, respectively, from a one-way ANOVA to assay statistical significance of the measured mean-value variation of a given variable between different cochlear locations for inner (IHC) and outer (OHC) hair cells, from two-tailed unpaired Student's *t*-tests with Welch’s correction to compare mean values of the variable between two groups of a given hair-cell type (IHC or OHC) with different characteristic frequencies (CF) or between the two cell types (OHC/IHC) when they are associated to the same characteristic frequency. The last entry provides the p-value to assay the statistical significance between the slopes of a weighted linear regression of the relation between the variable and the characteristic frequency of the hair cell. A bold font was used to help find statistically significant differences. The variables in the table correspond to the relative contribution $r$ and the absolute contribution $K_{\mathrm{GS}}$ of the gating springs to the hair-bundle stiffness, the contribution $K_{\mathrm{SP}}$ of the stereociliary pivots to the hair-bundle stiffness, the rotational stiffness $\kappa$ of a single stereocilium, and the stiffness $k_{\mathrm{GS}}$ of a single gating spring.
