# Supplementary material for: Stiffness and tension gradients of the hair cell’s tip-link complex in the mammalian cochlea
Source: eLife. 2019 Apr 1;8:e43473. doi: 10.7554/eLife.43473 (PMC6464607; doi:10.7554/eLife.43473)
Supplement: Figure 6—source data 1. — The table lists p-values resulting, respectively, from a one-way ANOVA to assay statistical significance of the measured mean-value variation of a given variable between different cochlear locations for inner (IHC) and outer (OHC) hair cells, from two-tailed unpaired Student's t-tests with Welch’s correction to compare mean values of the variable between two groups of a given hair-cell type (IHC or OHC) with different characteristic frequencies (CF) or between the two cell types (OHC/IHC) when they are associated to the same characteristic frequency. The last entry provides the p-value to assay the statistical significance between the slopes of a weighted linear regression of the relation between the variable and the characteristic frequency of the hair cell. A bold font was used to help find statistically significant differences. The variables in the table correspond to the negative hair-bundle movement ΔXCa, the corresponding increase in hair-bundle tension ΔT, and the maximal tension tmax in a single gating spring evoked by EDTA iontophoresis just before tip-link disruption. [file elife-43473-fig6-data1.docx]

|  | **ANOVA** | | **IHC** | | | | | |
| --- | --- | --- | --- | --- | --- | --- | --- | --- |
|  | IHC | OHC | 1-2 kHz | 1-4 kHz | 1-15 kHz | 2-4kHz | 2-15 kHz | 4-15 kHz |
| $\Delta X_{\mathrm{Ca}}$ | *p* =  1.9×10^-1^ | *p* =  4.6×10^-1^ | *p* =  6.8×10^-1^ | *p* =  9.5×10^-2^ | *p* =  4.1×10^-1^ | ****p* =**  **2.2×10^-2^** | *p* =  2.3×10^-1^ | *p* =  6.0×10^-1^ |
| $\Delta T$ | ******p* =**  **6.5×10^-6^** | *****p* =**  **5.1×10^-3^** | *p* =  6.4×10^-1^ | ******p* =**  **6.3×10^-4^** | *****p* =**  **5.9×10^-3^** | ******p* =**  **2.8×10^-4^** | *****p* =**  **4.2×10^-3^** | *p* =  4.0×10^-1^ |
| $t_{\max}$ | ******p* =**  **3.1×10^-6^** | ******p* =**  **7.2×10^-4^** | *p* =  5.8×10^-1^ | *****p* = 1.4×10^-3^** | *****p* =**  **1.8×10^-3^** | ******p* =**  **5.1×10^-4^** | ******p* =**  **7.7×10^-4^** | *p* =  1 |
|  | **OHC** | | | **OHC/IHC** | | | Gradient OHC *vs.*  gradient IHC | |
|  | 1-2 kHz | 1-4 kHz | 2-4 kHz | 1 kHz | 2 kHz | 4 kHz |  |  |
| $\Delta X_{\mathrm{Ca}}$ | *p* =  4.3×10^-1^ | *p* =  2.7×10^-1^ | *p* =  5.9×10^-1^ | *p* =  3.5×10^-1^ | *p* =  8.2×10^-1^ | *p* =  4.6×10^-1^ | *p* =  7.1×10^-2^ | |
| $\Delta T$ | ****p* =**  **4.9×10^-2^** | ****p* =**  **1.0×10^-2^** | *p* =  5.8×10^-2^ | *p* =  8.8×10^-1^ | *****p* =**  **5.5×10^-3^** | *p* =  2.7×10^-1^ | ****p* =  1.7×10^-2^** | |
| $t_{\max}$ | *p* =  1.3×10^-1^ | *****p* =**  **3.9×10^-3^** | ****p* =**  **2.8×10^-2^** | *p* =  8.4×10^-1^ | ****p* =  4.9×10^-2^** | *p* =  4.9×10^-1^ | ****p* =  2.2×10^-2^** | |

**Figure 6‒source data 1: Statistical significance.**

The table lists p-values resulting, respectively, from a one-way ANOVA to assay statistical significance of the measured mean-value variation of a given variable between different cochlear locations for inner (IHC) and outer (OHC) hair cells, from two-tailed unpaired Student's *t*-tests with Welch’s correction to compare mean values of the variable between two groups of a given hair-cell type (IHC or OHC) with different characteristic frequencies (CF) or between the two cell types (OHC/IHC) when they are associated to the same characteristic frequency. The last entry provides the p-value to assay the statistical significance between the slopes of a weighted linear regression of the relation between the variable and the characteristic frequency of the hair cell. A bold font was used to help find statistically significant differences. The variables in the table correspond to the negative hair-bundle movement $\Delta X_{\mathrm{Ca}}$, the corresponding increase in hair-bundle tension $\Delta T$, and the maximal tension $t_{\max}$ in a single gating spring evoked by EDTA iontophoresis just before tip-link disruption.
